# Renal injury after uninephrectomy in male and female intrauterine growth-restricted aged rats

**DOI:** 10.1371/journal.pone.0213404

**Published:** 2019-03-07

**Authors:** Ashley D. Newsome, Gwendolyn K. Davis, Osasu N. Adah, Norma B. Ojeda, Barbara T. Alexander

**Affiliations:** 1 Department of Physiology and Biophysics, University of Mississippi Medical Center, Jackson, Mississippi, United States; 2 Department of Pediatrics, University of Mississippi Medical Center, Jackson, Mississippi, United States; University Medical Center Utrecht, NETHERLANDS

## Abstract

Epidemiological studies report an inverse association between birth weight and risk for kidney disease that may differ between males and females, but studies investigating this association are limited. This study tested the hypothesis that male intrauterine growth-restricted offspring in a model of low birth weight induced by placental insufficiency in the rat exhibit enhanced renal injury in response to a persistent secondary renal insult while female growth-restricted offspring are protected. For this study, control offspring from sham-operated dams and growth-restricted offspring from reduced uterine perfusion dams underwent uninephrectomy or a sham procedure at 18 months of age. One month later, urinary markers of renal injury, renal function, and histological damage were measured. Results were analyzed using 2-way ANOVA. Male and female offspring were assessed separately. Proteinuria and urinary neutrophil gelatinase-associated lipocalin were significantly elevated in male growth-restricted offspring exposed to uninephrectomy when compared to male uninephrectomized control. Urinary kidney injury marker-1 was elevated in male uninephrectomized growth-restricted offspring relative to male sham growth-restricted but not to male uninephrectomized controls. Likewise, urinary neutrophil gelatinase-associated lipocalin was elevated in female uninephrectomized growth-restricted offspring but only when compared to female sham growth-restricted offspring. Markers of renal function including glomerular filtration rate and serum creatinine were impaired after uninephrectomy in female offspring regardless of birth weight. Histological parameters did not differ between control and growth-restricted offspring. Collectively, these studies suggest that both male and female growth-restricted offspring demonstrate susceptibility to renal injury following uninephrectomy; however, only male growth-restricted offspring exhibited an increase in renal markers of injury in response to uninephrectomy relative to same-sex control counterparts. These findings further suggest that urinary excretion of protein, kidney injury marker-1, and neutrophil gelatinase-associated lipocalin may be early markers of kidney injury in growth-restricted offspring exposed to a secondary renal insult such as reduction in renal mass.

## Introduction

Low birth weight (**LBW**), defined as less than 2500 grams in humans, is a well-established risk factor for hypertension [1–5], impaired glucose homeostasis [1, 6–10], and reduced nephron number [11,12]. Epidemiological studies also report that LBW individuals are more likely to develop chronic kidney disease (**CKD**) and end-stage renal disease [13–18]. Although the increased prevalence of hypertension and diabetes among LBW individuals contributes to this association, evidence suggests that these factors alone do not fully account for the greater risk of kidney disease [15, 19–20]. Several studies indicate that CKD risk may differ between LBW men and LBW women [20, 21]. Experimental studies also report that the age-related decline in renal function and injury differs between male and female offspring exposed to a developmental insult [22–27]. Likewise, numerous experimental models of LBW or intrauterine growth restriction (**IUGR**) report that male growth-restricted offspring exhibit elevated blood pressure during early adulthood while female littermates are protected [27–35]. Collectively, these studies demonstrate that adverse events during early life program enhanced risk for later chronic disease that may differ by sex. Yet, whether LBW or IUGR programs an enhanced vulnerability to a secondary renal insult in adulthood is not well defined. Additionally, whether the renal response to an additional renal insult in later life differs in males and females is also understudied.

Our laboratory uses a model of LBW or IUGR induced by a mechanical reduction of uterine blood flow in the pregnant rat leading to placental insufficiency [28]. In this model, baseline glomerular filtration rate (**GFR**) is normal in male growth-restricted offspring at 3 months of age [28] and in female growth-restricted at 12 months of age [36] despite an elevated blood pressure in male and female growth-restricted offspring relative to same-sex controls at these respective ages. Ojeda reported that male growth-restricted offspring at 6 months of age exhibit a reduction in GFR and an increase in renal tubular injury 2 hours after exposure to a mild, acute renal insult of 15 minutes of renal ischemia followed by reperfusion, an insult that does not affect male controls [37]. Although this study indicated that IUGR programs susceptibility to a secondary renal insult in male growth-restricted offspring not observed in control counterparts, whether IUGR increases susceptibility to a renal insult that persists for a longer period has not yet been determined.

Evidence suggests that LBW individuals may be more vulnerable to a reduction in renal mass such as the decrease of nephrons that occurs over a lifespan or the loss of renal mass experienced with organ donation. Berglund et al. found a negative association between birth weight and albuminuria in human kidney donors [38], and Schachtner and Reinke report a reduced GFR and increased proteinuria in LBW living kidney donors [39]. Jedrzejko and colleagues did not observe a relationship between birth weight and proteinuria after kidney donation, but they did find a greater prevalence of hypertension after removal of one kidney in LBW donors [40]. Thus, the aim of this study was to determine whether adult growth-restricted offspring exhibit enhanced susceptibility to a persistent renal insult via uninephrectomy. The results of this study indicate that male growth-restricted offspring are vulnerable to a persistent renal insult relative to their male control counterparts. Female growth-restricted offspring who underwent uninephrectomy differ only compared to their female growth-restricted sham littermates.

## Materials and methods

Timed pregnant primigravid Sprague-Dawley dams (Envigo, Madison, WI) at 10–12 weeks of age were received on day 13 of gestation and housed individually in a temperature-controlled room at 23°C with a 12:12 hour light/dark cycle. They were fed a global soy protein-free extruded rodent diet (Envigo) to avoid the effect of dietary phytoestrogens and were given water *ad libitum*. All procedures were done in compliance with the National Institutes of Health guidelines for use and care of animals, and the protocol was approved by the Institutional Animal Care and Use Committee at the University of Mississippi Medical Center (Protocol Number: 0878E). All surgeries were performed under isoflurane anesthesia with carprofen provided for post-surgical pain control, and all efforts were made to minimize suffering. A total of 12 sham-operated and 14 reduced uterine perfusion pressure dams were used to generate all offspring utilized in the following study.

### Induction of intrauterine growth restriction

On day 14 of gestation, dams were randomly assigned to undergo reduced uterine perfusion pressure surgery to generate growth-restricted offspring or a sham surgery to produce control offspring, as previously described [28, 41]. Briefly, silver clips were placed on the uterine arteries and lower abdominal aorta to reduce blood flow to the developing pups. The uterine horn was visualized in sham-operated dams. Animals delivered naturally on days 21–22 of gestation, and control and growth-restricted pups were weighed within 12 hours of birth. After delivery, male and female offspring were culled to a litter size of eight per dam to ensure comparable feeding, and offspring were weaned at three weeks of age.

### Induction of a secondary renal insult

Male and female offspring were randomly selected for study at 18 months of age and for inclusion in the sham-operated (**Sham**) or uninephrectomized (**UNI-X**) groups (S1 Table). In UNI-X groups, the right renal vascular pedicle was ligated before removal of the right kidney. For sham surgeries, the right renal artery was visually observed before closing without further manipulation. Four groups were studied in both males and females: control sham, control UNI-X, growth-restricted sham, and growth-restricted UNI-X.

### Detection of urinary and hematologic markers of kidney injury

24-hour metabolism cage studies were performed to measure urinary markers of renal injury prior to sham surgery or uninephrectomy and four weeks after surgery. Proteinuria was determined by pyragollol red urine assay (Quantimetrix, Redondo Beach, CA). Urinary excretion of KIM-1 and NGAL were determined by ELISA (Abcam, Cambridge, MA and R&D Systems, Minneapolis, MN). Blood was collected four weeks after surgery immediately after GFR measurement at the time of tissue harvest, and serum creatinine and blood urea nitrogen (**BUN**) were measured from these samples using the VET AXCEL Chemistry Analyzer (Alfa Wassermann, West Caldwell, NJ) located in the Analytical and Assay Core.

### Measurement of glomerular filtration rate

At 19 months of age, four weeks post sham surgery or uninephrectomy, GFR was measured by transcutaneous fluorescein isothiocyanate (**FITC**)-sinistrin clearance in conscious, unrestrained rats as described previously with small modifications detailed here [42–43]. A NIC-Kidney transducer device (Mannheim Pharma and Diagnostics GmbH, Mannheim, Germany) was secured to the animal using a rodent harness (Instech Laboratories Inc, Plymouth Meeting, PA). A single bolus injection of 7.5 mg/kg of FITC-sinistrin (Fresenius-Kabi, Linz, Austria) was given via jugular venous catheter followed by a 0.06 mL saline flush in animals instrumented the previous day. The excretion half-life was used to calculate GFR using a semi-empirical conversion factor according to the kinetic model detailed by Friedemann et al.: GFR [mL/min/100 g BW) = 21.33 [mL/100 g BW] / t_½_ (FITC-S) [min]. [44]

### Histological assessment of kidney injury

At the end of the experiment, the left kidney was harvested and hemisected along its long axis, fixed by submersion in 10% buffered formalin for 48 hours, then transferred to 70% ethanol for storage. Kidneys were processed in paraffin, sectioned, and stained with hematoxylin and eosin or Masson’s trichrome for light microscopy. All histological analysis of renal injury was assessed on blinded samples, and images were viewed and captured on a Nikon Eclipse 50i microscope with a Nikon DS-Qi1Mc camera (Nikon Instruments Inc., Melville, NY). Thirty glomeruli were measured at 400x magnification using NIS-Elements Basic Research 2.30 software (Nikon Instruments Inc., Melville, NY) to determine glomerular area. The measurement function within the software was manually calibrated prior to data collection. Glomerular area was obtained using the area interactive measurement function by drawing a circle outlining each glomerulus using the on-screen cursor. Uniformly round glomeruli were chosen for measurement to reduce variability due to differences in plane of sectioning. All measurements were averaged for each section to obtain the mean glomerular area for each kidney.

For determination of glomerular injury score (**GIS**), fifty glomeruli per section were scored by visual inspection at 400x magnification. A score of 1–4 was assigned based on degree of mesangial matrix expansion, collapsed capillaries, and uniformity of Bowman’s capsule, with GIS 1 representing <25% of the glomerulus containing evidence of sclerotic lesions, GIS 2 representing 25–50%, GIS 3 representing 50–75%, and GIS 4 representing >75% of the glomerulus containing evidence of sclerosis including few to no obviously patent capillaries and apparent obliteration of Bowman’s space. From the 50 individual scores, an average GIS score was calculated for each kidney.

NIS-Elements BR was also used for semi-quantitative analysis of percent interstitial fibrosis and detection of tubular protein casts. The average percentage of injury was calculated from six images per section captured at 40x magnification from the renal medulla. To determine interstitial fibrosis, areas of collagen deposition were stained blue with Masson’s trichrome. In the software program, a one point threshold tool was used to manually mark distinct areas of blue staining. The selected areas were then used by the system to detect and highlight similar points. Once the observer used an on-screen magnifying function to ensure the system captured most visible areas of collagen staining, the threshold was set and utilized as a reference to measure staining on each kidney section. The software utilized the threshold to isolate areas of blue staining and count the number of objects (groups of neighboring pixels) within the highlighted area. Percent fibrosis was calculated from the number of highlighted objects divided by the total number of objects within a field of measurement, and individual measurements from each slide were averaged to provide a value for each sample. The same threshold technique was used to measure protein casts, but in this case images were captured under green fluorescence using the Nikon Intensilight C-HGFI (Nikon Instruments Inc., Melville, NY) illumination hardware attached to the microscope and camera described above. Protein casts appeared bright red on hematoxylin and eosin stained sections, and these areas were used to establish the threshold and semi-quantitative measurements.

### Statistical analysis

Data were analyzed using GraphPad 7.02 software. To investigate the interaction for IUGR and uninephrectomy, we performed 2-way ANOVA with Tukey’s multiple comparisons test. The statistical tests were two-sided tail. Urinary markers of renal injury (proteinuria, KIM-1, and NGAL), renal function (glomerular filtration rate, serum creatinine, and BUN), and histological injury (glomerular area, GIS, interstitial fibrosis, and tubular protein casts) were included as the outcome variables, with IUGR and uninephrectomy as the exposures of interest. Since this study was not powered to study sex-differences, male and female offspring were evaluated separately.

Power analysis was performed separately in males and females using proteinuria and NGAL as the primary outcomes. Sample size was calculated with SAS system (http://euclid.psych.yorku.ca/cgi/power.pl) for a 2X2 ANOVA with an effect size of 1.25, confidence level of 0.05, and power of 0.80 for males and females separately in each group. When sample sizes are not reached due to high attrition rates, the lower limit for power analysis will be set to 0.60. Statistical significance was set for alpha ≤ 0.05. Each variable was additionally analyzed after normalizing by body weight, which did not result in any changes in significance.

## Results

### Litter size, birth weight, and body weight

Birth weight was significantly reduced in growth-restricted offspring relative to same-sex controls (Table 1). Litter sizes varied from four to twelve pups per sham litter and six to fourteen pups per RUPP litter. The average number of pups per litter was similar in sham-operated relative to RUPP dams (7.8 ± 0.8 versus 9.4 ± 0.8, p-value = 0.17). Body weight remained lower in male growth-restricted offspring with uninephrectomized growth-restricted offspring demonstrating a significantly lower body weight compared to same-sex control and sham growth-restricted offspring at 19 months of age (Table 1). However, female growth-restricted offspring experienced catch-up growth by three months of age and had no significant difference in body weight at 19 months of age. Left kidney weight at the time of harvest was compared to determine whether the remnant kidney in uninephrectomized offspring demonstrated hypertrophy. In males, uninephrectomized growth-restricted offspring had a significantly greater remnant kidney weight compared to Sham growth-restricted offspring, but not compared to controls (Table 1). In females, both control and growth-restricted offspring who underwent uninephrectomy had a significantly greater remnant kidney weight compared to sham-operated offspring when this was normalized by body weight.

**Table 1:** Group characteristics: Birth weight, body weight, and kidney weight.

Experimental Groups	Birth Weight, g	Body weight at 19 months of Age, g	Left Kidney Weight, g	Left Kidney weight to Body Weight Ratio, mg/g
**Males**				
Control Sham n = 7	5.78±0.13	537±12	1.89±0.11	3.54±0.23
Control UNI-X n = 7	6.05±0.12	537±20	2.17±0.13	4.10±0.34
IUGR Sham n = 5	5.18±0.08#	521±7	1.47±0.09	2.82±0.17
IUGR UNI-X n = 9	5.09±0.10#	447±15*#	2.24±0.15*	5.05±0.38*
**Females**				
Control Sham n = 7	6.03±0.08	309±9	0.94±0.03	3.07±0.14
Control UNI-X n = 7	6.25±0.12	275±4	1.07±0.03	3.90±0.08*
IUGR Sham n = 10	5.09±0.15#	286±12	0.87±0.04	3.04±0.09
IUGR UNI-X n = 7	5.23±0.13#	296±13	1.08±0.06*	3.68±0.17*

Results are mean ± SEM.

*P<0.05 vs sham counterpart;

^#^P<0.05 vs control counterpart. IUGR, intrauterine growth restricted; UNI-X, uninephrectomized.

### Urinary and serum markers of renal injury

**Fig 1:**
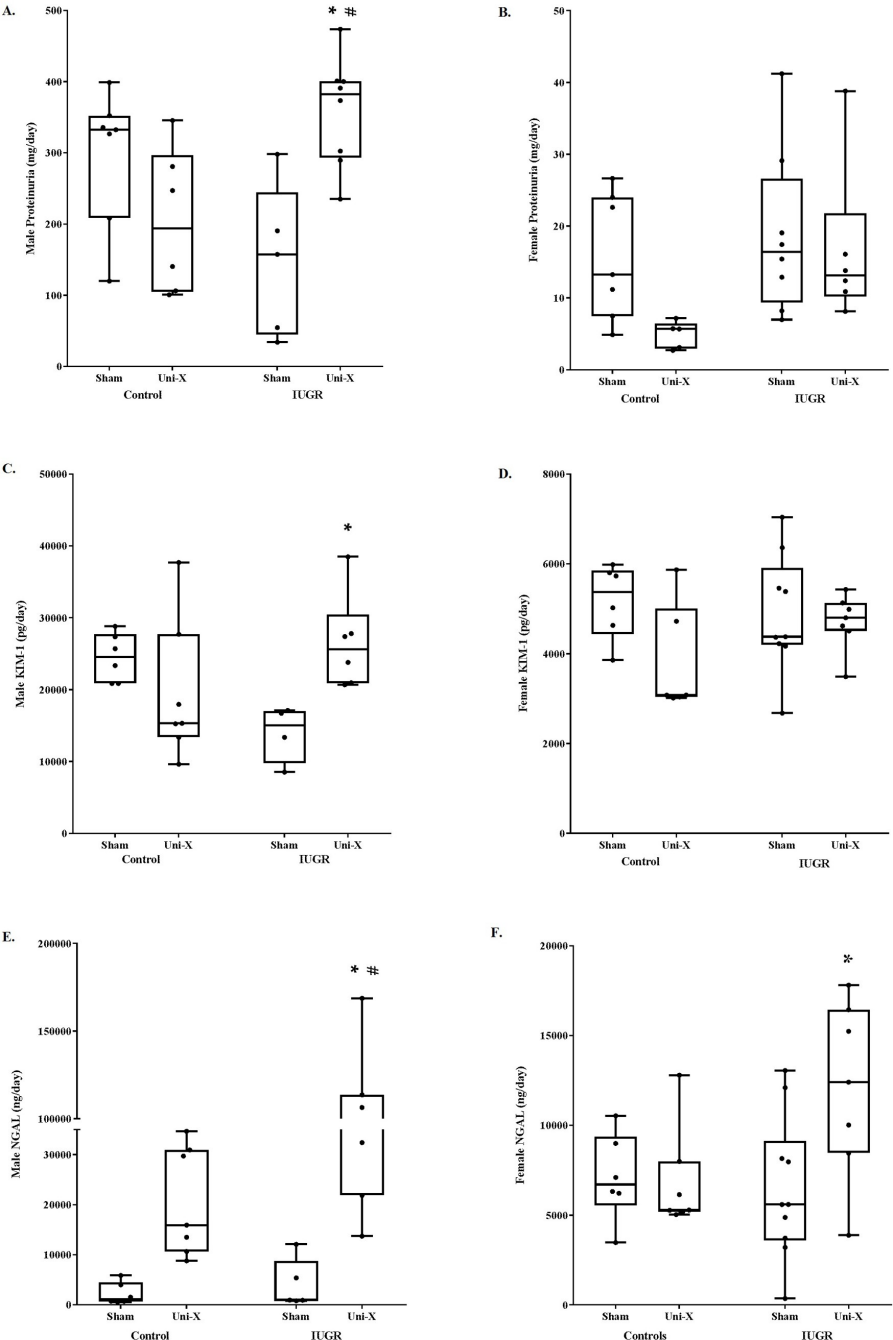
Urinary markers demonstrate greater renal injury in growth-restricted offspring exposed to uninephrectomy compared to same-sex control and sham offspring. Male data are shown in the left-sided panel, and female data are shown on the right. (**A-B**) Proteinuria is elevated in male but not female growth-restricted offspring after uninephrectomy. (**C-D**) Urinary excretion of kidney injury marker-1, a specific marker of proximal tubular injury, is elevated only in male uninephrectomized growth-restricted offspring compared to male sham growth-restricted offspring. (**E-F**) Urinary excretion of NGAL, a marker of acute kidney injury, is elevated in both male and female uninephrectomized growth-restricted offspring compared to sham growth-restricted, but only male growth-restricted have increased NGAL excretion compared to uninephrectomized controls. *P<0.05 vs sham counterpart, ^#^P<0.05 versus control counterpart. IUGR, intrauterine growth restricted; KIM-1, kidney injury marker-1; NGAL, neutrophil gelatinase-associated lipocalin; UNI-X, uninephrectomized. Proteinuria sample sizes, males: control sham = 7, control UNI-X = 6, IUGR sham = 5, IUGR UNI-X = 8. Proteinuria sample sizes, females: control sham = 7, control UNI-X = 5, IUGR sham = 8, IUGR UNI-X = 6. KIM-1 sample sizes, males: control sham = 6, control UNI-X = 7, IUGR sham = 4, IUGR UNI-X = 6. KIM-1 sample sizes, females: control sham = 6, control UNI-X = 6, IUGR sham = 9, IUGR UNI-X = 7. NGAL sample sizes, males: control sham = 6, control UNI-X = 7, IUGR sham = 5, IUGR UNI-X = 7. NGAL sample sizes, females: control sham = 6, control UNI-X = 7, IUGR sham = 10, IUGR UNI-X = 7.

At 19 months of age, proteinuria was significantly greater in male uninephrectomized growth-restricted offspring compared to male sham growth-restricted offspring and uninephrectomized controls, while females showed no difference in proteinuria between groups (Fig 1A and 1B). Male growth-restricted offspring exposed to uninephrectomy also had higher urinary KIM-1 excretion compared to sham-operated male growth-restricted offspring, but female groups did not differ (Fig 1C and 1D). Both male and female uninephrectomized growth-restricted offspring had increased urinary NGAL compared to their same-sex sham growth-restricted counterparts (Fig 1E and 1F), but only male uninephrectomized growth-restricted had increased urinary NGAL compared to male uninephrectomized controls (Fig 1E).

### Renal function

**Fig 2:**
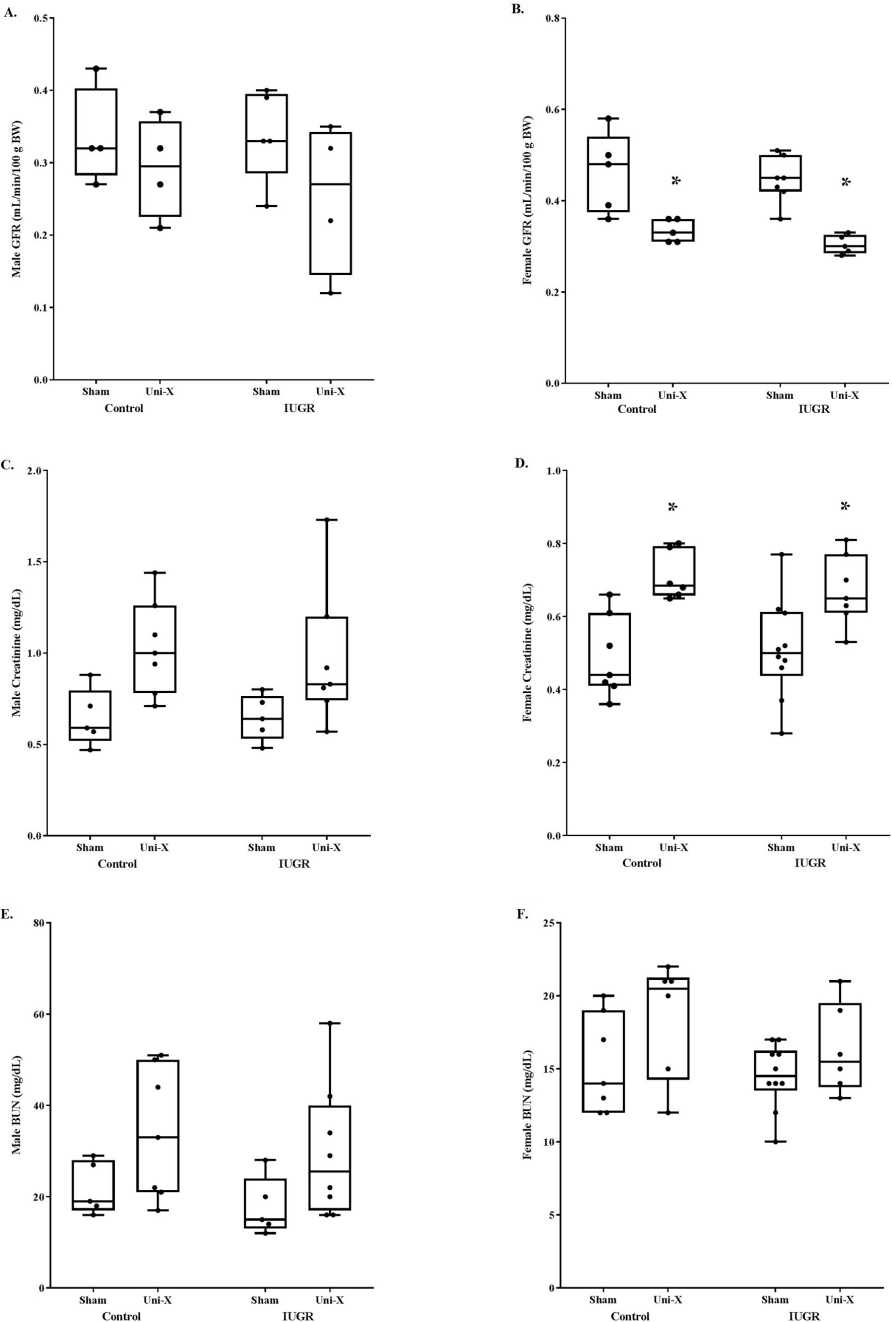
Glomerular filtration rate is reduced in female offspring regardless of birth weight whereas renal function is not altered by uninephrectomy in male rats. Male data are shown in the left-sided panel, and female data are shown on the right. (**A-B**) GFR does not differ in male offspring, but GFR is significantly decreased in female control and growth-restricted offspring after uninephrectomy relative to sham counterparts. (**C-D**) Serum creatinine does not differ in male offspring, but is increased in female control and growth-restricted offspring after uninephrectomy relative to sham counterparts. (**E-F**) Blood urea nitrogen is similar between all groups. All groups were compared to same-sex counterparts. *P<0.05 vs sham counterpart. BUN, blood urea nitrogen; GFR, glomerular filtration rate; IUGR, intrauterine growth restricted; UNI-X, uninephrectomized. GFR sample sizes, males: control sham = 4, control UNI-X = 4, IUGR sham = 5, IUGR UNI-X = 4. GFR sample sizes, females: control sham = 5, control UNI-X = 5, IUGR sham = 7, IUGR UNI-X = 5. Creatinine sample sizes, males: control sham = 5, control UNI-X = 7, IUGR sham = 5, IUGR UNI-X = 7. Creatinine sample sizes, females: control sham = 7, control UNI-X = 6, IUGR sham = 10, IUGR UNI-X = 7. BUN sample sizes, males: control sham = 5, control UNI-X = 7, IUGR sham = 5, IUGR UNI-X = 8. BUN sample sizes, females: control sham = 7, control UNI-X = 6, IUGR sham = 10, IUGR UNI-X = 6.

At 19 months of age, four weeks after exposure to uninephrectomy, female uninephrectomized offspring had a significant reduction in GFR (Fig 2B) and a significant increase in serum creatinine (Fig 2D) compared to same-sex sham counterparts. This was evident in both control and growth-restricted offspring. Male offspring did not demonstrate a change in these parameters after uninephrectomy (Fig 2A and 2C), and BUN was not altered in male or female offspring regardless of birth weight (Fig 2E and 2F).

### Histological evidence of renal injury

Neither glomerular area, glomerular injury score, or degree of protein cast formation differed between groups in either males or females in the current study (S2 Table). Interstitial fibrosis differed only between female control sham and control uninephrectomized offspring (S1 Table). Representative images of glomerulosclerosis, interstitial fibrosis, and protein casts that were used for scoring and establishing thresholds for semi-quantitative measurements are shown (S1 Fig), as well as example histology slides from each of the uninephrectomized groups (S2–S4 Figs).

## Discussion

The results of this study indicate that male growth-restricted offspring exposed to uninephrectomy in later life had a significant increase in proteinuria and urinary excretion of NGAL compared to uninephrectomized male controls and sham-operated male growth-restricted counterparts. In addition, male growth-restricted offspring who underwent uninephrectomy had increased KIM-1 compared to sham-operated male growth-restricted offspring. NGAL was also significantly increased in female growth-restricted offspring exposed to uninephrectomy relative to sham-operated growth-restricted females, but exhibited no differences in urinary KIM-1 or proteinuria. Together, these results suggest that growth-restricted offspring and male growth-restricted offspring in particular, are more susceptible to a persistent renal insult such as uninephrectomy in later life. Measures of renal function and markers of histological renal injury did not differ after uninephrectomy between same-sex control and growth-restricted offspring. Therefore, these data suggest that urinary markers may serve as early biomarkers of renal injury before functional or histological changes occur in offspring exposed to intrauterine growth restriction.

### Urinary markers of renal injury

While proteinuria is a long-established marker for renal injury, urinary NGAL and KIM-1 are less commonly used molecules that are proposed as biomarkers for the detection of kidney disease [45]. Studies concerning the efficacy of these molecules as renal biomarkers are mixed. NGAL is a lipocalin protein released from injured renal tubular epithelial cells after an acute insult [46]. It is produced most abundantly in the distal nephron, and the molecule is useful because urinary NGAL can be detected prior to changes in renal functional parameters. NGAL has been proposed as an indicator for both diagnosis and to determine the prognosis of acute kidney injury, as it is elevated within hours from the time of insult and hours to days before changes in serum creatinine are detected [47]. It is not clear how long urinary NGAL remains elevated and therefore how efficacious this molecule is as a marker of chronic renal injury is not yet known. The current study involves an abrupt decrease in renal mass at the time of uninephrectomy followed by a prolonged exposure to glomerular hyperfiltration that leads to progressive renal injury. This month of hyperfiltration is much longer than previous acute studies that have investigated renal effects over hours, but may still be too brief to demonstrate truly chronic effects relative to the human condition of renal donation.

KIM-1 is a glycoprotein produced by renal proximal tubular cells in response to injury [48]. Although it is also produced in the settings of acute renal injury, unlike NGAL, KIM-1 is characterized as an indicator of chronic disease as well as acute renal injury. While NGAL can be associated with systemic inflammation, urinary KIM-1 is more specific for renal injury. KIM-1 has been found to predict progression to nephropathy and greater renal dysfunction in diabetic patients [49–50]. This may be due to its location in the proximal tubules, which have a greater energetic demand in the presence of hyperglycemia and glomerular hyperfiltration in the diabetic patient. Both urinary NGAL and KIM-1 have potential prognostic value in diabetic patients [51].

Lobato et al. reported that NGAL but not urinary KIM-1 was correlated with later progression to advanced renal disease [52]. However, Seibert and colleagues reported that NGAL and KIM-1 were not useful biomarkers in CKD [53]. Likewise, a review investigating the prognostic value of tubular injury markers had mixed results, with neither KIM-1 nor NGAL providing additional value beyond GFR and albuminuria in a broad population of CKD patients [54]. However, in this and other studies, urinary NGAL did display predictive value when elevated in patients prior to detection of proteinuria, suggesting that tubular injury may develop prior to glomerular damage and could therefore be a very early indicator of renal disease [55].

### Changes in renal function and histology in various models of developmental insult

While urinary markers of renal injury differed significantly between control and growth-restricted offspring in the current study, renal function and histological renal injury did not differ between control and growth-restricted offspring at 19 months of age regardless of sex. Previous studies from our laboratory reported no difference in baseline GFR between control and growth-restricted offspring in the absence of a secondary renal insult [28, 36]. In these studies, GFR was measured at 3 months in males and 12 months in females. Woods et al. also reported no difference in GFR at 20 weeks of age in male offspring born from dams exposed to low protein diet during gestation (**LPD**, 8.5–9% casein versus 18–19% in controls [33]. However, male LPD offspring exhibited an increase in glomerular size indicative of hyperfiltration. Glomerular area did not differ in the present study at 19 months of age, but it is not known whether glomerular size is different between control and growth-restricted offspring in earlier life. In addition, it is not known whether the male LPD offspring which experience early glomerular hypertrophy would develop reduced renal function with age. Because the renin-angiotensin system is critical for proper renal development, blockade of this system is utilized as another method of developmental insult [56]. In a rat model of developmental programming induced by exposure to an angiotensin receptor antagonist (**ARA**), Loria et al. reported that GFR is normal at 3 months of age, but is reduced by 11 months of age in male but not female ARA offspring compared to controls [25, 26]. Thus, studies by Loria et al. suggest that exposure to a developmental insult leads to a greater reduction in GFR over time. Collectively, these studies suggest that differences in renal function in adult offspring exposed to a developmental insult may vary due to timing or severity of the model and the age at time of study.

### IUGR and secondary renal insult

Few studies have investigated the effect of IUGR on the response to a secondary renal insult. This represents an important gap in the literature because evidence suggests that LBW individuals have a lower functional reserve leading to subclinical renal injury that only manifest as clinical signs of kidney disease after a secondary insult. Plank et al. found that LPD offspring have a more severe course of IgA nephropathy with greater histological evidence of renal injury than their control counterparts when both were exposed to anti-Thy-1.1 antibody from 8 to 10 weeks of age [57]. In a study by Zimanyi et al., male LPD offspring were infused with advanced glycation end-products for 4 weeks to mimic the effects of diabetes [58]. The LPD group had greater expression of profibrotic compounds when compared to controls at 24 weeks of age; yet, no overt signs of renal damage were observed. These studies demonstrate differential responses to a persistent renal injury between control offspring and those exposed to a developmental insult, but as in our current study, they do not detect a clear reduction in GFR or an increase in histological injury after the secondary insult that is specific to the LBW or IUGR offspring. However, more work is needed to investigate longer periods of persistent injury as this might better reflect conditions such as chronic diabetes or a permanent reduction in renal mass after kidney donation in LBW individuals.

### Renal susceptibility in males and females

We are one of few groups to investigate the response to developmental programming of renal injury or markers of kidney disease in both males and females. Previously, Boubred et al. studied sex-specific renal effects in two different models of LBW: maternal LPD or maternal treatment with betamethasone (**BET**) during gestation [23]. Despite a similar reduction in birth weight in both models, only male BET offspring had a lower creatinine clearance than control offspring at 12 months of age and higher proteinuria at 15 months, while male and female LPD offspring and female BET showed normal renal function. Glomerulerosclerosis was significantly higher in both male and female BET offspring compared to controls at 22 months of age but remained normal in male and female LPD offspring relative to controls. When Black et al. studied LPD offspring at 32 and 100 weeks of age, both male control and male LPD offspring had a significant decline in GFR with aging, whereas female controls maintained normal renal function with aging resulting in a GFR that was significantly greater than male controls at 100 weeks of age [22]. However, this protection was lost in LPD females suggesting that a developmental insult alters the protective effect of the female sex on renal risk. Finally, Loria et al. also studied both sexes in their model of ARA induced via exposure to angiotensin II antagonism during the nephrogenic period [26]. Male ARA offspring had a greater decline in GFR and an age-related increase in proteinuria not found in female ARA offspring. Collectively, these studies provide evidence that developmental programming of renal risk is dependent on the model of developmental insult and is influenced by the sex of the offspring.

### Limitations

This study was designed to examine the effect of a secondary renal insult with persistent effects such as uninephrectomy in growth-restricted offspring in adulthood. Kidney donors range in age from newborn to 65 with a median age of 41 years [59]. By 18 months of age, the rat is equivalent to approximately 45 years of human life [60]. Thus, in our study the timing of uninephrectomy was equivalent to viable human donation. However, the length of time required to observe changes in renal function in response to a secondary insult with using experimental models is unclear. Other studies in rodent models have shown that the degree of renal hypertrophy and changes in GFR in response to uninephrectomy even in adults who were not exposed to a developmental insult is sex-specific [61], with uninephrectomized males demonstrating increased blood pressure and more severe renal lesions relative to uninephrectomized females at 18 months after uninephrectomy [62]. Another study that provides an estimate of relative age in humans compared to rodents calculates that the time of study at one month post uninephrectomy was equivalent to about 4 years of human life [60]. One study in LBW humans showed evidence of renal damage at 12, 36, and 60 months after donation, while another studied LBW humans at 10–15 years after kidney donation [38–39]. Hence, our study may not have fully recapitulated the long-term influence of renal donation on later renal health, not allowing for the subsequent long-term effect of hyperfiltration on renal function and morphology. Findings in the current study are also limited in detecting sex differences because the sample size was not large enough to allow direct comparison of males to females.

### Significance

Despite these limitations, this study is one of only a few to investigate the renal response to a loss of renal mass in adult growth-restricted offspring. Data from our study show that male growth-restricted offspring have a greater degree of renal injury demonstrated in multiple parameters. However, males and females exhibited significant interactions between IUGR and uninephrectomy in the case of enhanced NGAL urinary excretion. This finding suggests that markers of renal function and renal injury may be tailored to the type of subjects in which they will be utilized. “One size fit all” may not apply to subjects exposed to early insults and susceptible to programming of adverse long-term outcomes.

The current data, alongside the previously described studies, indicate that different models of developmental insult result in varying levels of renal dysfunction and patterns of kidney injury in response to a secondary renal hit later in life. As the causes of LBW are diverse, different animal models are useful because each represents a particular subset of the LBW population. This has implications for both epidemiological investigations and clinical practice because the cause of abnormal fetal growth may be as important as birth weight itself in determining which individuals are at greater risk for renal injury and disease. Separating patients by cause of developmental insult may strengthen the effects of previously reported correlations in epidemiological studies, and clinicians may benefit from observing not only a patient’s birth weight, but also specific details of their perinatal history. Sex as a biological variable should also be considered as a modifier of renal risk and chronic disease. Another potential application of the current study is that urinary markers such as NGAL and KIM-1 in addition to microalbuminuria may be used as screening tools for later kidney disease in LBW individuals, although more study is necessary to explore the efficacy of these molecules as biomarkers.

### Conclusion

In summary, the type, severity, and timing of adverse events during early development can affect long-term renal outcomes following a secondary renal insult later in life. Individuals exposed to adverse developmental influences may require tailored tests to recognize renal risk with sex included as a consideration. Future experimental studies will benefit from investigating secondary renal insults initiated at a younger age with longer follow-up, and in response to more severe injury. Collectively, these studies will facilitate a better understanding of the mechanisms that contribute to enhanced renal risk and provide better preventative approaches and treatment strategies among the LBW population. Additionally, biomarkers such as NGAL and KIM-1 may serve as improved early markers of renal injury in studies determining the efficacy of interventions for preventing or reversing kidney disease in LBW individuals.

## Supporting information

S1 Table Number of pups utilized from each litter. Littermates from dams that were not randomly selected at earlier ages for other studies were aged up to 18 months of age. When possible, same-sex littermates were randomly selected to undergo either the sham or uninephrectomized procedure. However, the number of viable, same-sex offspring from a single litter was limited by 18 months of age due to the high incidence of spontaneous tumor development within the colony, an observation that has been extensively reported by others [63, 64, 65, 66]. S1 Table provides the breakdown of litter source per study group.
(DOCX)

S2 Table Histological evidence of renal injury at one month after uninephrectomy. GIS, glomerular injury score; IUGR, intrauterine growth restricted; UNI-X, uninephrectomized.Results are mean ± SEM. *P<0.05 vs Sham counterpart.
(DOCX)

S1 Fig Representative images used for scoring and semi-quantitative measurements of renal injury. **A.** Depiction of glomerular injury scores 1–4. **B and C**. Black arrows demonstrate concentrated areas of interstitial fibrosis, while white arrows demonstrate concentrated areas of protein cast formation.
(TIF)

S2 Fig Representative image of glomeruli from each uninephrectomized group. IUGR, intrauterine growth restricted; UNI-X, uninephrectomized.
(TIF)

S3 Fig Representative image of interstitial fibrosis from each uninephrectomized group. IUGR, intrauterine growth restricted; UNI-X, uninephrectomized.
(TIF)

S4 Fig Representative image of protein casts from each uninephrectomized group. IUGR, intrauterine growth restricted; UNI-X, uninephrectomized.
(TIF)

## Abbreviations

ARA: angiotensin II receptor antagonist exposure
BET: betamethasone exposure
BUN: blood urea nitrogen
CKD: chronic kidney disease
IUGR: intrauterine growth restricted
FITC: fluorescein isothiocyanate
GIS: glomerular injury score
GFR: glomerular filtration rate
KIM-1: kidney injury marker-1
LBW: low birth weight
LPD: low protein diet
NGAL: neutrophil gelatinase-associated lipocalin
UNI-X: uninephrectomized
