# Supplementary material for: Renal injury after uninephrectomy in male and female intrauterine growth-restricted aged rats
Source: PLoS One. 2019 Mar 7;14(3):e0213404. doi: 10.1371/journal.pone.0213404 (PMC6405063; doi:10.1371/journal.pone.0213404)
Supplement: S1 Table — Littermates from dams that were not randomly selected at earlier ages for other studies were aged up to 18 months of age. When possible, same-sex littermates were randomly selected to undergo either the sham or uninephrectomized procedure. However, the number of viable, same-sex offspring from a single litter was limited by 18 months of age due to the high incidence of spontaneous tumor development within the colony, an observation that has been extensively reported by others [63, 64, 65, 66]. S1 Table provides the breakdown of litter source per study group. (DOCX) [file pone.0213404.s001.docx]

| **S1 Table.** Number of pups utilized from each litter | |
| --- | --- |
| **Experimental Groups** | **Offspring were generated from:** |
| **Males** | |
| Control Sham | 5 dams total: 1-2 offspring per dam |
| Control UNI-X | 5 dams total: 1 offspring from 4 of the dams, 3 offspring from the final dam |
| IUGR Sham | 3 dams total: 1-2 offspring per dam |
| IUGR UNI-X | 4 dams total: 2 offspring each from 3 of the dams, 3 offspring from the final dam |
| **Females** | |
| Control Sham | 5 dams total: 1-2 offspring per dam |
| Control UNI-X | 4 dams total: 1 offspring each from 3 of the dams, 4 offspring from the final dam |
| IUGR Sham | 7 dams total: 1-2 offspring per dam |
| IUGR UNI-X | 5 dams total: 1-2 offspring per dam |
