# Supplementary material for: Renal injury after uninephrectomy in male and female intrauterine growth-restricted aged rats
Source: PLoS One. 2019 Mar 7;14(3):e0213404. doi: 10.1371/journal.pone.0213404 (PMC6405063; doi:10.1371/journal.pone.0213404)
Supplement: S2 Table — GIS, glomerular injury score; IUGR, intrauterine growth restricted; UNI-X, uninephrectomized.Results are mean ± SEM. *P<0.05 vs Sham counterpart. (DOCX) [file pone.0213404.s002.docx]

| **S2 Table.** Histological Markers of Renal Injury | | | | |
| --- | --- | --- | --- | --- |
| **Experimental Groups** | **Glomerular Area, μm^2^** | **Glomerular Injury Score, 0-4** | **Interstitial Fibrosis, %** | **Protein Cast, %** |
| **Males** | | | | |
| Control Sham, n=7 | 19763±1028 | 2.174±0.163 | 6.587±0.363 | 18.023±2.692 |
| Control UNI-X, n=7 | 16861±596 | 1.649±0.095 | 5.327±1.357 | 12.084±2.468 |
| IUGR Sham, n=5 | 19559±1109 | 2.236±0.133 | 5.598±0.762 | 7.266±2.013 |
| IUGR UNI-X, n=9 | 20490±1641 | 2.142±0.169 | 6.693±0.995 | 13.204±2.929 |
| **Females** | | | | |
| Control Sham, n=4 | 11841±192 | 1.320±0.151 | 6.618±1.267 | 2.805±1.423 |
| Control UNI-X, n=6 | 11911±289 | 1.430±0.033 | 5.021±0.649* | 3.863±0.825 |
| IUGR Sham, n=5 | 12502±454 | 1.485±0.017 | 3.173±0.428 | 4.185±1.822 |
| IUGR UNI-X, n=4 | 12637±1025 | 1.455±0.033 | 3.423±0.623 | 2.078±0.631 |
