# Supplementary figures and images for: Renal injury after uninephrectomy in male and female intrauterine growth-restricted aged rats
Source: PLoS One. 2019 Mar 7;14(3):e0213404. doi: 10.1371/journal.pone.0213404 (PMC6405063; doi:10.1371/journal.pone.0213404)

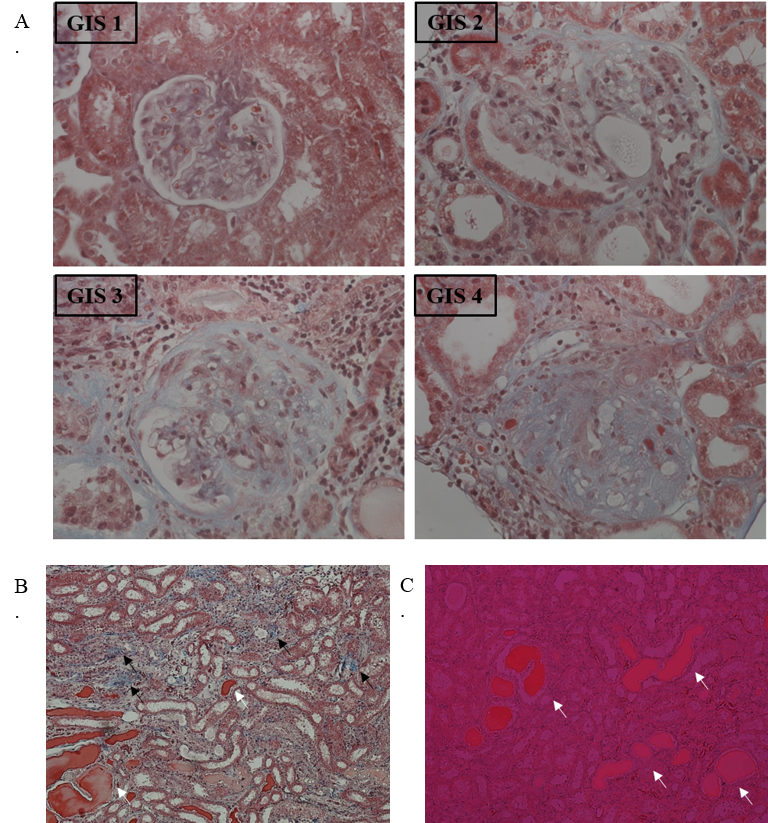

Supplement: S1 Fig — A. Depiction of glomerular injury scores 1–4. B and C. Black arrows demonstrate concentrated areas of interstitial fibrosis, while white arrows demonstrate concentrated areas of protein cast formation. (TIF) [file pone.0213404.s003.tif]

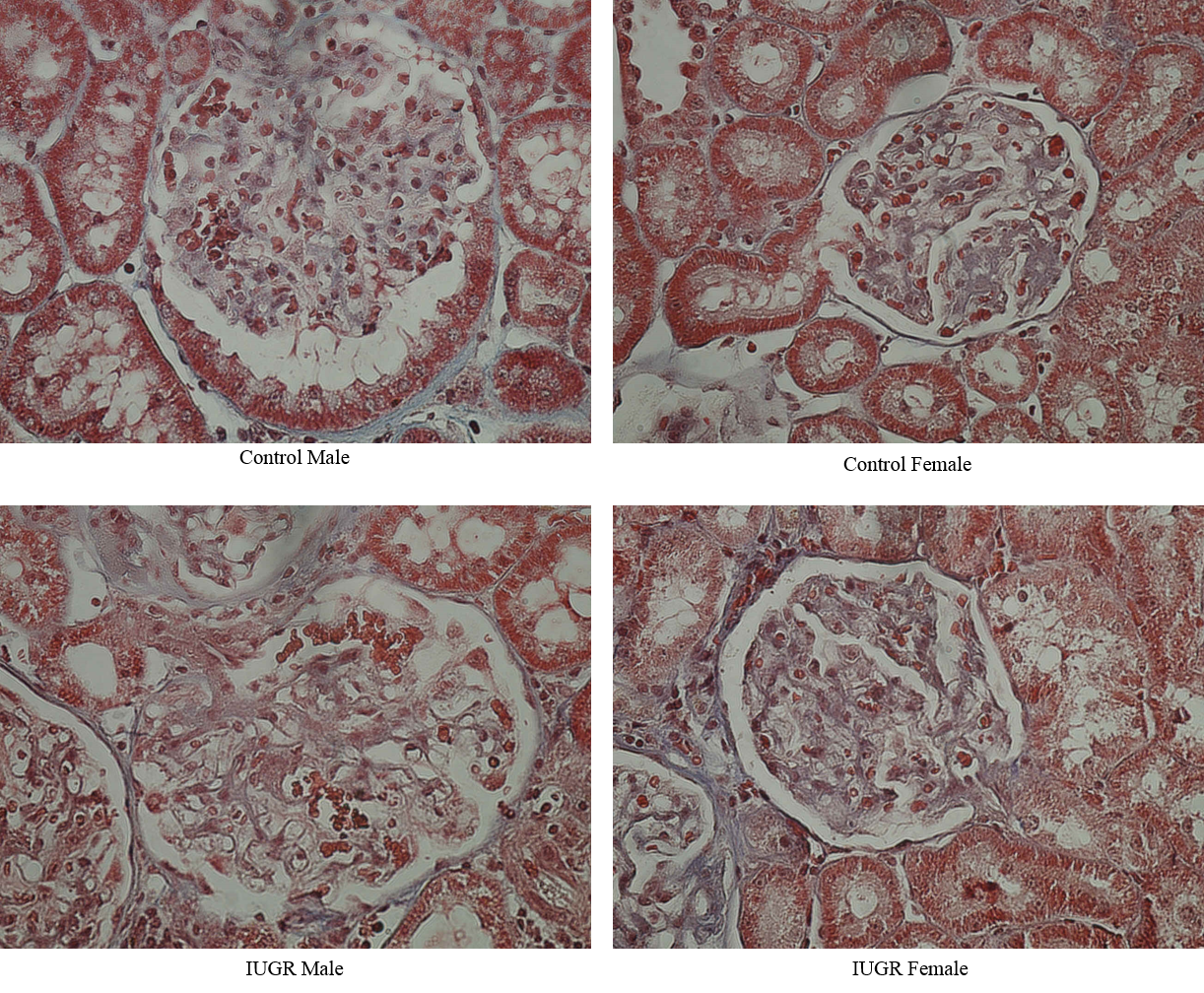

Supplement: S2 Fig — IUGR, intrauterine growth restricted; UNI-X, uninephrectomized. (TIF) [file pone.0213404.s004.tif]

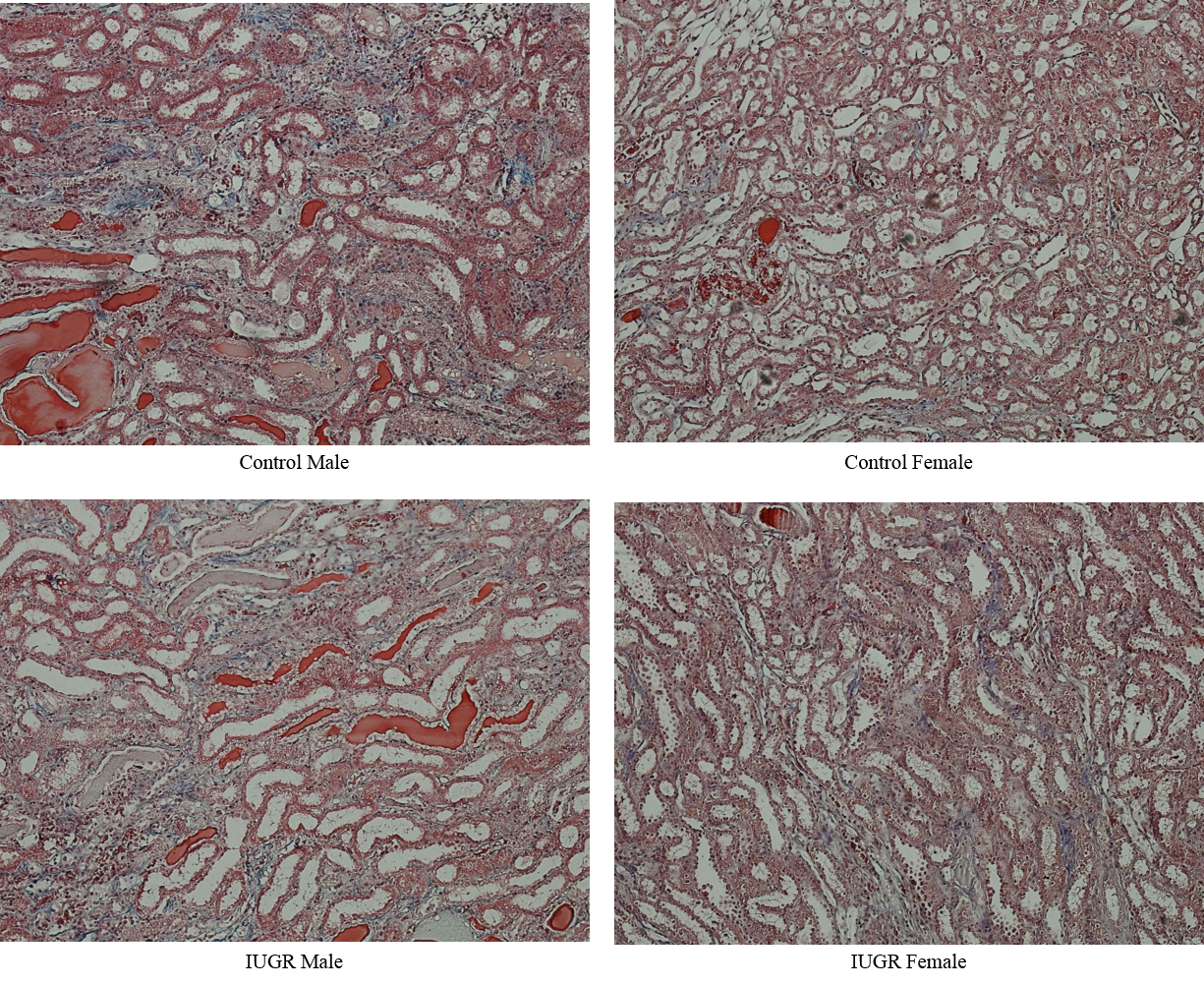

Supplement: S3 Fig — IUGR, intrauterine growth restricted; UNI-X, uninephrectomized. (TIF) [file pone.0213404.s005.tif]

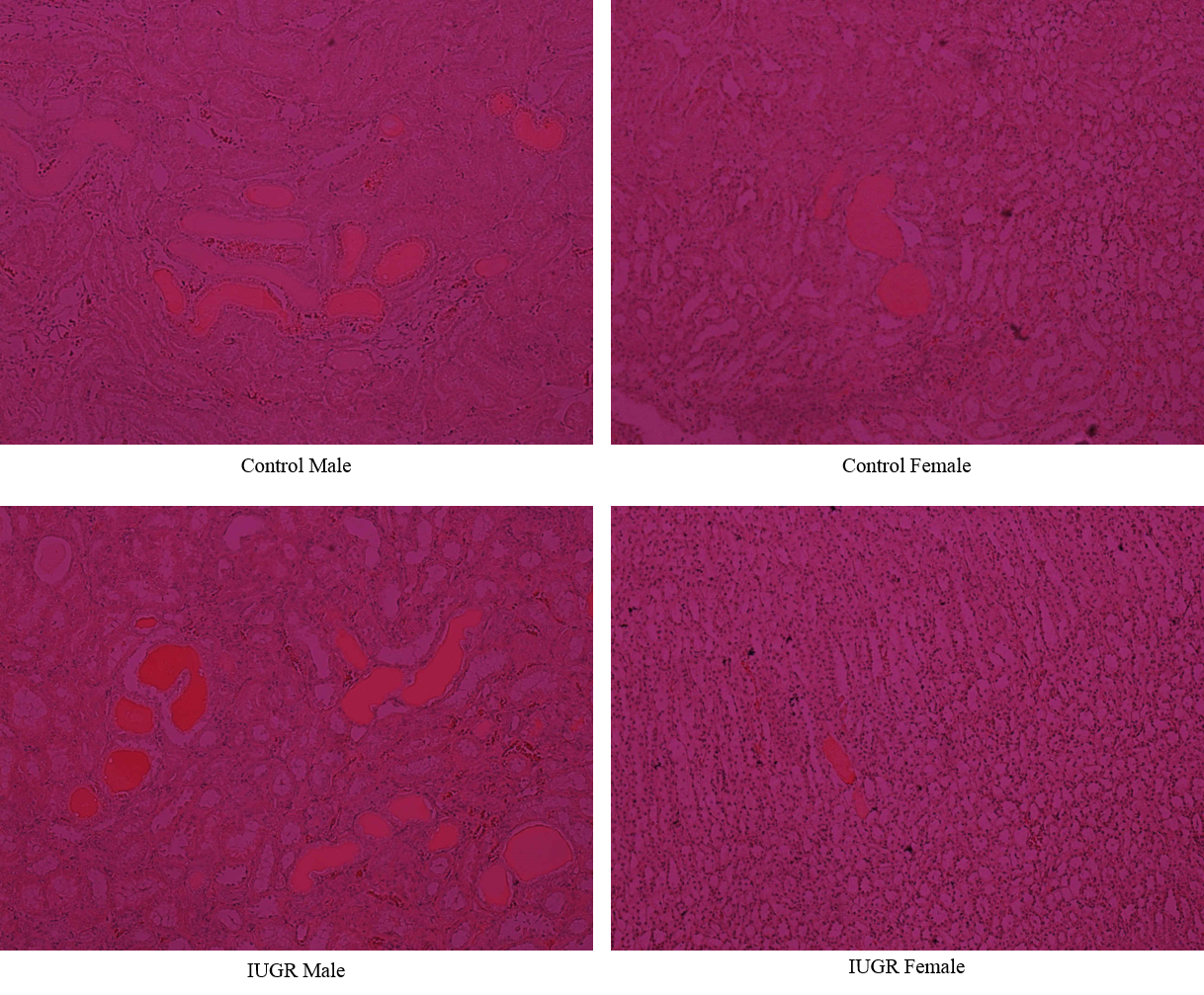

Supplement: S4 Fig — IUGR, intrauterine growth restricted; UNI-X, uninephrectomized. (TIF) [file pone.0213404.s006.tif]
